# Unsuspected diversity and multiple origins of the frog legs imported to Switzerland for human consumption, as determined by DNA barcoding and morphology

**DOI:** 10.1007/s00114-025-01968-2

**Published:** 2025-02-13

**Authors:** Sylvain Dubey, Sébastien Pellaud, Samuel Furrer, Christophe Dufresnes

**Affiliations:** 1 Department of Ecology and Evolution, University of Lausanne, 1015 Lausanne, Switzerland; 2 Chemin Des Vergers 7, 1941 Vollèges, Switzerland; 3 Schweizer Tierschutz STS, Dornacherstrasse 101, 4018 Basel, Switzerland; 4 Institut de Systématique, Evolution, Biodiversité (ISYEB), Muséum National d’Histoire Naturelle, CNRS, Sorbonne Université, EPHE-PSL, Université Des Antilles, 55 Rue Buffon, CP 51, 75005 Paris, France

**Keywords:** Amphibian, Biological invasion, Food production, International trade

## Abstract

The frog leg industry relies on a global, largely underregulated market with potentially important ecological impact such as the uncontrolled harvest of declining wild populations and the introduction of invasive species. Here, we inferred the taxonomic nature and geographic origins of frog legs imported to Switzerland by DNA barcoding. Out of 34 samples, we retrieved eight distinct lineages attributed to five species from four genera, namely *Hoplobatrachus rugulosus* from Vietnam, *Fejervarya cancrivora* from Indonesia (invasive on several Pacific islands), two phylogeographic lineages of *Limnonectes macrodon* from Western and Central Java, *L. kadarsani* from eastern Indonesia, and three phylogeographic lineages of *Pelophylax ridibundus* from northern and central southern Turkey (invasive in Western Europe). Only the first two species were correctly declared, which is particularly problematic to track down harvests of the declining and geographically restricted *Limnonectes* taxa. In this respect, we show that the three Asian genera can be reliably distinguished by basic measurements of the frog legs, which could be used in future forensic controls. Our study calls for more stringent international regulations of the frog trade, including shipment monitoring to document the relative abundance of harvested species and ensure the sustainability of their wild populations.

**Supplementary Information:**

The online version contains supplementary material available at 10.1007/s00114-025-01968-2.

## Introduction

Due to the environmental and ecological impact of the food industry, as well as reported cases of malpractice by producers and vendors, public awareness toward the composition and origin of food products is growing (e.g., Ayaz et al. 2006; Brodmann et al. 2001; Kyrova et al. 2016; Pappalardo and Ferrito 2015; Shears 2010; Teletchea et al. 2005). These issues are topical for the frog legs’ business, which trades several million tons of meat representing billions of specimens worldwide annually, through an abstruse, largely underregulated market (Altherr et al. 2011; Auliya et al. 2016, 2023a; Gratwicke et al. 2009; Warkentin et al. 2009). Amphibian meat is typically no longer declared in the UN Comtrade international trade database (Gerson 2012), even though the demand is increasing. Frogs are considered both a valuable alternative nutritional source in developing countries such as in Africa and Asia (Mohneke et al. 2010; Neang 2010), and as a delicacy in North America and Europe (Altherr et al. 2011). Despite the rapid growth of frog farms (Aabedi et al. 2014; Helfrich et al. 2009; Moreira et al. 2013) and progress in aquaculture research (Ding et al. 2015; Gui and Zhu 2012; Martínez et al. 2004; Neveu 2009), most traded frog meat still originates from wild populations (Auliya et al. 2023b).

The over-harvesting of wild populations can have dramatic consequences, such as rapid regional declines and the disruption of ecosystem balance (Auliya et al. 2023a, 2023b; Mohneke and Rödel 2009; Ohler and Nicolas 2017; Veith et al. 2000). For instance, the collapse of frog species in India and Bangladesh such as *Phrynoderma hexadactylum* and *Hoplobatrachus tigerinus* due to harvest for the international trade in the 1960s and the 1980s led to the propagation of the agricultural pests normally regulated by these species, an absence that was compensated by higher pesticide use and the associated environmental costs (Auliya et al. 2023a; Oza 1990). Moreover, frog farms often import live animals from overseas, which can contribute to biological invasions due to frequent escape and release (e.g., Dubois 1983; Yu et al. 2015). For instance, the diversity of introduced *Pelophylax* populations in Western Europe coincides with the diversity found among captive-bred stocks (Bellati et al. 2023).

With nearly 50,000 tons corresponding to 1–2 billion individuals in the 2000s, Europe remains one of the biggest importers of frog legs (Altherr et al. 2011, 2022; Auliya et al. 2023b; Kusrini and Alfold 2006). Following historical interdictions to commercially harvest local populations as well as export bans from several tropical countries such as India (Ohler and Nicolas 2017), the main suppliers of the European frog leg market are now Indonesia and Vietnam and, to a smaller extent, Turkey and Albania (Altherr et al. 2022; Auliya et al. 2023b). Different species of frogs are harvested in these regions, namely from the family Dicroglossidae in Southeast Asia (genera *Hoplobatrachus*, *Limnonectes*, *Fejervarya*) and from the family Ranidae in southeastern Europe and Asia minor (genus *Pelophylax*, mainly the marsh frog *P. ridibundus* sensu Dufresnes et al. 2024, see also Frost 2025). A large proportion of these exports continues to originate from the wild and conservation concerns are growing. For instance, up to a billion specimens were collected every year in Indonesia in the early 2000s (Kusrini 2005; Kusrini and Alfold 2006), and these activities are potentially responsible for the decline of the widely commercialized species *L. macrodon* (Ohler and Nicolas 2017). Likewise, monitoring and modelling studies predicted that the exploitation of *Pelophylax* in Turkey is unsustainable, as the export demands exceed the demographic capacity of populations (Çiçek et al. 2021).

The monitoring and management of harvested populations on the one hand and the path towards appropriate international agreements and trading regulations on the other hand require accurate species identification of the exported frog legs (Auliya et al. 2016, 2023a, 2023b; Ohler and Nicolas 2017; Veith et al. 2000). However, while most amphibian species can be distinguished based on their morphology and coloration, frog legs are skinned, packed, and deep-frozen without their body prior to their transportation, which make their identification challenging to verify (Warkentin et al. 2009). Molecular methods such as DNA barcoding are thus necessary for accurate species identification (e.g., Ohler and Nicolas 2017) and can in turn validate the diagnosticity of measurable anatomical characters. As such, DNA barcoding has revealed numerous discrepancies between declarations and nature of frog leg exports, which can lead to a gross under- or over-representation of the diversity of harvested species. For instance, Veith et al. (2000) showed that frog legs imported from Indonesia to the European Union (EU) were listed as four different species, although they singly represent *F. cancrivora*. Likewise, frog legs sold in French supermarkets are declared as *L. macrodon* even though they also essentially represent *F. cancrivora* (Ohler and Dubois 2017). In Germany, some commercial frog legs are properly labelled (*H. rugulosus* from Vietnam), but other similarly include *F. cancrivora* in packages labelled *L. macrodon* (Dittrich et al. 2017).

The Swiss frog leg market presumably represents about 150 tons corresponding to 7.5–10 million individuals every year, including 450,000 individuals (30 tons) transported live and collected in the wild, mainly from Turkey (Altherr et al. 2022). The latter are euthanized and prepared in the country, an activity that is yet not considered sufficiently important to be subjected to a particular authorization (Anonymous 2010). Nevertheless, live imports for the frog leg industry are believed to have contributed to the introduction and current invasions of multiple exotic lineages of *P. ridibundus* across Western Europe (Bellati et al. 2023; Dufresnes et al. 2024; Holsbeek et al. 2008), which established and spread over Switzerland during the second half of the twentieth century (Dufresnes et al. 2018). Although still poorly understood, the ecological impact of the marsh frog invasions is worrisome due to their predation risk on native animals (Pille et al. 2021, 2023), the deregulation of native *Pelophylax* communities through hybridization and competition (Holsbeek and Jooris 2010), their broad habitat tolerance (Denoël et al. 2022) and potential performance under future climate condition (Padilla et al. 2023), and as potential vector of diseases such as chytridiomycosis (Baláž et al. 2014; Jakóbik et al. 2024).

Given the conservation challenges, both foreign and domestic, accurate scientific information on the nature and origin of the frog legs traded in Switzerland will be important to inform stakeholders and debate future regulations of this trade. To this end, here we used DNA barcoding to determine the species imported by several independent suppliers of frog legs involving distinct geographic origins and modalities (frozen legs vs. live frogs transformed locally). The genetic diversity retrieved among the Swiss frog leg samples was considered in the framework of the species’ documented diversity, both in the wild and among documented frog farms and other frog leg imports in neighboring countries. In addition, we measured morphological characters and searched for diagnostic criteria to distinguish between the different dicroglossid genera identified among the frog legs imported from southeast Asia.

## Material and methods

### Genetic analyses

Thirty-four frog legs were obtained from five different importing and distributing companies (*n* = 2–20 individuals each), therein labelled brands A–E for confidentiality. Their origins and species were given as Vietnam (*Hoplobatrachus rugulosus*), Indonesia (*Fejervarya cancrivora*), and Turkey (*Rana esculenta* [= *Pelophylax esculentus*]). For all but one brand (A), frog legs were obtained in two separated batches acquired between several months’ intervals. Brands A–D sell frog legs imported frozen, while brand E imports live frogs to Switzerland to be transformed locally. Total cellular DNA was extracted from pieces of tissues using the DNeasy Blood & Tissue Kit (Qiagen).

For all but one sample, a ~ 550 bp fragment of 16S rRNA mitochondrial gene was amplified by polymerase chain reaction (PCR) using the primers 16SAR and 16SBR (Simon et al. 1994). 16S has been used extensively to characterize anuran diversity, especially in Asia. PCR amplifications were conducted in 25 µL volumes, including 10.625 µL of H_2_O, 2.5 µL of Qiagen buffer (10 ×), 2.25 µL of MgCl_2_, 1.25 µL of each primer (10 mM), 1 µL of dNTPs (10 mM), 1 µL of betaine (5 M), 0.125 µL Taq polymerase, and 5 µL of template DNA. The thermocycling profile included 35 cycles of 30″ at 94 °C, 30″ at 50 °C, and 60″ at 72 °C. For samples identified as *Pelophylax*, we further amplified a ~ 340 bp fragment of the mitochondrial gene NADH dehydrogenase subunit 3 (*ND3*), which distinguishes among closely related lineages of *P. ridibundus* (e.g., Akın et al. 2010; Bellati et al. 2023; Dufresnes et al. 2024). PCR amplifications were carried out with the primers ND3L and ND3H (Plötner et al. 2008) following the same conditions as for 16S. Amplicons were sequenced by Sanger technology in the reverse direction (16SBR and ND3H).

Sequences were aligned and trimmed to up to 527 bp (16S) and 338 bp (*ND3*) in MEGA 11 (Tamura et al. 2021), uploaded on GenBank under accessions PV013548-PV013580 (16S) and PV012986–PV012989 (*ND3*), and unique haplotypes were identified visually. To identify the maternal lineage and corresponding taxon of each haplotype, we matched them against the NCBI GenBank database using BLAST (Zhang et al. 2000) with the megablast algorithm with default settings. For the 16S haplotypes which closest hit corresponds to dicroglossid taxa, we built reference sequence sets by mining all the GenBank sequences featuring less than 3% of divergence (i.e., percentage identity) and gathered information on their taxonomic identity and geographic origins from the metadata available on GenBank and/or their associated publications. For the 16S and *ND3* haplotypes matching *Pelophylax*, we considered the haplotype datasets and associated metadata compiled for these genes based on thousands of DNA barcoding sequences by Dufresnes et al. (2024). For each dataset, we visualize the diversity and relative divergence of haplotypes by building phylogenetic networks of uncorrected p-distances with SplitsTree 4.18.3 (Huson and Bryant 2006) and mapped localities of geo-referenced sequences with QGIS 3.24.3.

### Morphological analyses

For frog legs imported from southeast Asia, which represent different dicroglossid genera (see Results), we searched for informative anatomical criteria by measuring the length of the tibia (TIB), the length of the femur (FEM), the length of the urostyle (URO), and the wet weight (WEI). Variation among these characters was visualized by a principal component analysis (PCA) computed with the R package FactoMineR (Le et al. 2008) and by boxplots of the ratios TIB/FEM, FEM/URO and TIB/URO computed with the R package ggplot2 (Wickham 2016). To statistically assess differences in the general shape of the frog legs, we conducted a Multivariate Analysis of Variance (MANOVA) on all four characters. We then test differences for each of the three ratios separately by Kruskal–Wallis tests (R package stats). To assess whether frog legs could be accurately assigned to their taxon with this character set, we performed a linear discriminant analysis (LDA) with JMP Pro 17 (SAS Institute, Cary, NC).

## Results

### Genetic analyses

Out of 33 frog legs sequenced, 12 unique 16S haplotypes labelled SFL01–12 were retrieved. These are representative of five currently recognized species from four genera, namely *H. rugulosus*, *L. macrodon*, *L. kadarsani*, *F. cancrivora*, and *P. ridibundus* (Table 1, Supporting Information).

**Table 1:** Information on the analyzed frog legs commercially imported to Switzerland. Tibia length (TIB), femur length (FEM), and urostyle length (URO) are in mm; weight (WEI) in g

Code	Brand	Declared origin	Declared species	Identified taxon (lineage)	16S Accession	16S haplo	*ND3* Accession	*ND3* haplo	TIB	FEM	URO	WEI
3.1	A	Vietnam	*Hoplobatrachus rugulosus*	*Hoplobatrachus rugulosus* (S)	PV013548	SFL01	-	-	34.0	34.0	24.5	28.6
3.2	A	Vietnam	*Hoplobatrachus rugulosus*	*Hoplobatrachus rugulosus* (S)	PV013549	SFL01	-	-	34.5	34.0	23.0	26.2
2.1	B	Vietnam	*Hoplobatrachus rugulosus*	*Hoplobatrachus rugulosus* (S)	PV013550	SFL01	-	-	40.3	39.1	24.8	46.2
2.2	B	Vietnam	*Hoplobatrachus rugulosus*	*Hoplobatrachus rugulosus* (S)	PV013551	SFL01	-	-	43.0	38.4	24.5	45.3
4.1	B	Vietnam	*Hoplobatrachus rugulosus*	*Hoplobatrachus rugulosus* (S)	PV013552	SFL01	-	-	46.0	45.0	26.5	52.4
4.2	B	Vietnam	*Hoplobatrachus rugulosus*	*Hoplobatrachus rugulosus* (S)	PV013553	SFL01	-	-	40.0	35.6	24.2	47.1
1.1.8	C	Indonesia	*Fejervarya cancrivora*	*Fejervarya cancrivora*	PV013554	SFL04	-	-	39.9	36.6	27.5	30.1
1.1.9	C	Indonesia	*Fejervarya cancrivora*	*Fejervarya cancrivora*	PV013555	SFL02	-	-	39.4	33.2	27.6	30.7
1.1.10	C	Indonesia	*Fejervarya cancrivora*	*Fejervarya cancrivora*	PV013556	SFL02	-	-	40.3	38.3	30.4	28.2
1.1.15	C	Indonesia	*Fejervarya cancrivora*	*Fejervarya cancrivora*	PV013557	SFL02	-	-	37.2	34.6	31.8	26.2
1.1.22	C	Indonesia	*Fejervarya cancrivora*	*Fejervarya cancrivora*	PV013558	SFL06	-	-	38.1	32.6	24.4	23.3
1.1.23	C	Indonesia	*Fejervarya cancrivora*	*Fejervarya cancrivora*	PV013559	SFL02	-	-	37.7	34.6	22.7	27.6
1.1.27.2	C	Indonesia	*Fejervarya cancrivora*	*Fejervarya cancrivora*	PV013560	SFL06	-	-	35.5	27.4	21.1	19.8
5.1.3	C	Indonesia	*Fejervarya cancrivora*	*Fejervarya cancrivora*	PV013561	SFL02	-	-	41.3	35.1	32.2	29.7
5.1.8	C	Indonesia	*Fejervarya cancrivora*	*Fejervarya cancrivora*	PV013562	SFL02	-	-	39.1	34.5	23.3	25.4
5.1.14	C	Indonesia	*Fejervarya cancrivora*	*Fejervarya cancrivora*	PV013563	SFL02	-	-	38.4	33.0	30.2	24.7
5.1.21	C	Indonesia	*Fejervarya cancrivora*	*Fejervarya cancrivora*	PV013564	SFL05	-	-	36.6	33.6	25.2	19.8
5.1.22	C	Indonesia	*Fejervarya cancrivora*	*Fejervarya cancrivora*	PV013565	SFL03	-	-	36.9	33.8	27.2	23.5
1.1	C	Indonesia	*Fejervarya cancrivora*	*Fejervarya cancrivora*	PV013566	SFL02	-	-	42.0	38.5	32.0	24.0
5.1.13	C	Indonesia	*Fejervarya cancrivora*	*Limnonectes kadarsani*	PV013567	SFL10	-	-	41.7	39.7	26.9	28.8
5.1	C	Indonesia	*Fejervarya cancrivora*	*Limnonectes macrodon* (C)	PV013572	SFL09	-	-	46.5	45.0	29.5	27.1
1.1.7	C	Indonesia	*Fejervarya cancrivora*	*Limnonectes macrodon* (W)	PV013568	SFL08	-	-	47.1	43.2	29.9	38.0
5.1.19	C	Indonesia	*Fejervarya cancrivora*	*Limnonectes macrodon* (W)	PV013569	SFL08	-	-	42.0	39.3	25.7	31.3
5.1.29	C	Indonesia	*Fejervarya cancrivora*	*Limnonectes macrodon* (W)	PV013570	SFL08	-	-	43.7	41.2	27.6	36.5
1.2	C	Indonesia	*Fejervarya cancrivora*	*Limnonectes macrodon* (W)	PV013571	SFL08	-	-	41.5	37.5	23.0	19.6
5.2	C	Indonesia	*Fejervarya cancrivora*	*Limnonectes macrodon* (W)	PV013573	SFL08	-	-	41.0	40.0	25.5	21.6
6.1	D	Indonesia	*Fejervarya cancrivora*	*Fejervarya cancrivora*	PV013574	SFL02	-	-	35.5	31.5	24.5	14.0
6.2	D	Indonesia	*Fejervarya cancrivora*	*Fejervarya cancrivora*	PV013575	SFL07	-	-	35.0	31.5	27.5	14.8
7.1	D	Indonesia	*Fejervarya cancrivora*	*Fejervarya cancrivora*	PV013576	SFL03	-	-	34.5	32.0	24.5	12.0
7.2	D	Indonesia	*Fejervarya cancrivora*	*Fejervarya cancrivora*	PV013577	SFL02	-	-	38.0	32.5	26.5	14.7
8.1	E	Turkey	*Rana esculenta*	*Pelophylax r.* cf. *ridibundus* (F)	PV013578	16S.026	PV012986	ND3.066	**-**	**-**	**-**	**-**
8.2	E	Turkey	*Rana esculenta*	*Pelophylax r.* cf. *ridibundus* (K)	PV013579	16S.036	PV012987	ND3.196	**-**	**-**	**-**	**-**
9.1	E	Turkey	*Rana esculenta*	*Pelophylax r.* cf. *ridibundus* (J)	-	-	PV012988	ND3.197	**-**	**-**	**-**	**-**
9.2	E	Turkey	*Rana esculenta*	*Pelophylax r.* cf. *ridibundus* (K)	PV013580	16S.036	PV012989	ND3.127	**-**	**-**	**-**	**-**

**Fig. 1:**
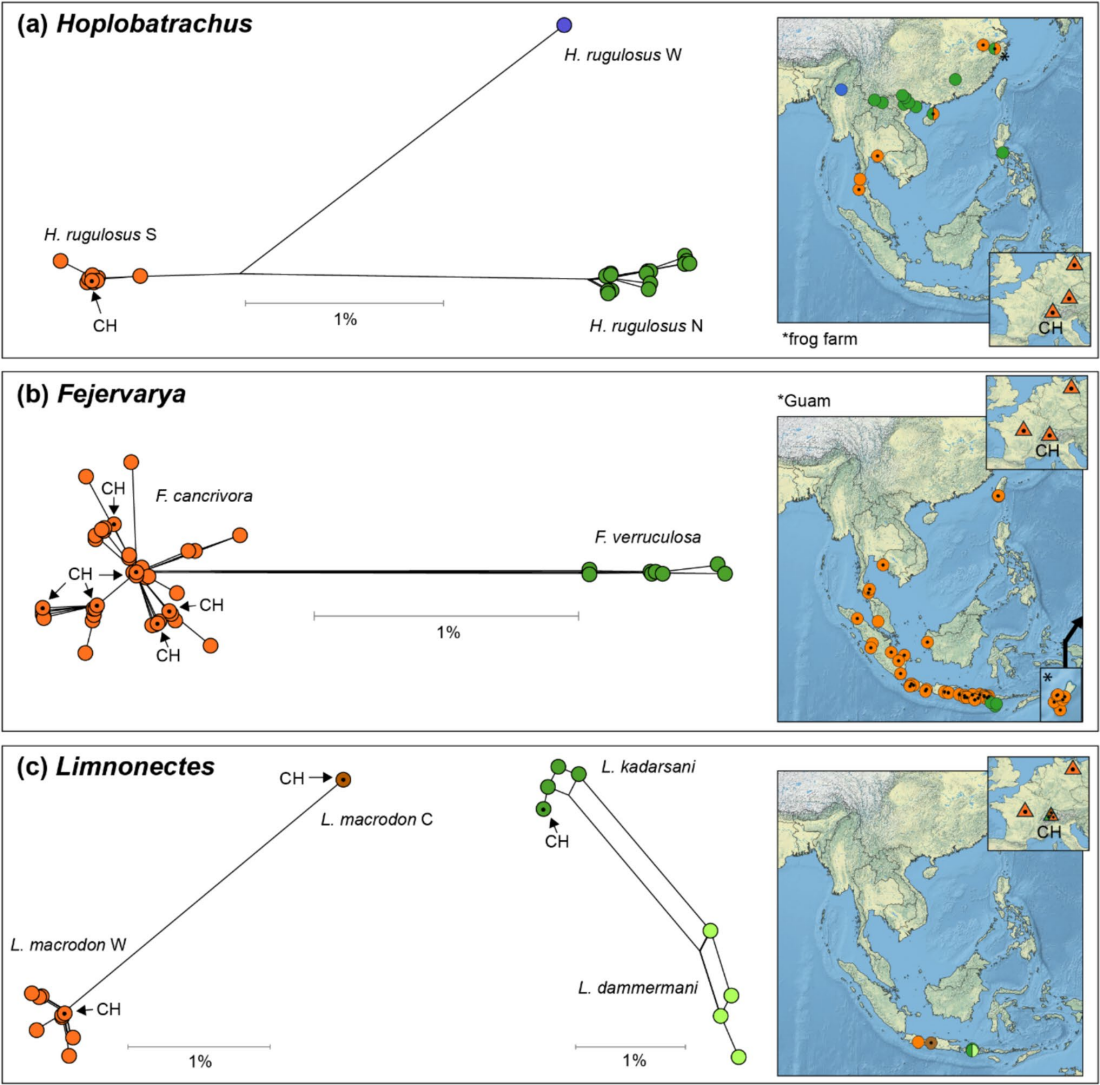
Phylogenetic divergence (networks) and geographic origins (maps) of the dicroglossid 16S sequences related to the Swiss frog leg samples (CH), separately for genus *Hoplobatrachus* (**a**), *Fejervarya* (**b**), and *Limnonectes* (**c**). Dots emphasize the haplotypes retrieved in the Swiss frog legs and where these haplotypes were previously found in natural populations in Asia (circles) and among other frog leg imports in Europe (triangles)

The 16S sequences of brands A and B, who declared their products as *H. rugulosus*, all consist of a single haplotype (SFL01) matching that species (Table 1). The phylogenetic network of sequences closely related to our samples (< 3% of sequence divergence) emphasizes the high mitochondrial diversity of the populations presently considered *H. rugulosus*, which involves three distinct phylogeographic lineages (Fig. 1a). SFL01 belongs to the southern lineage of *H. rugulosus* (orange in Fig. 1a), which has been retrieved in Thailand and southern and eastern China, where it was detected in the wild and in a frog farm (Yu et al. 2015). SFL01 is also the only haplotype retrieved among the frog legs belonging to that species in Asian buffets and supermarkets in Germany (Dittrich et al. 2017; Spielmann et al. 2018).

The 16S sequences of brands C and D, who declared their products as *F. cancrivora*, consist of nine haplotypes attributed to three distinct species (Table 1). Thirteen frog legs from brand C and all frog legs from brand D correspond to *F. cancrivora*, namely six haplotypes (SFL02–07; three new, three previously sequenced) that are broadly representative of the 16S diversity of that species across Indonesia, the south of mainland southeast Asia, but also introduced ranges such as Taiwan and Guam (Wostl et al. 2016), as well as frog legs sequenced in French and German supermarkets (Ohler and Nicolas 2017; Dittrich et al. 2017; orange in Fig. 1b). The closest relative of *F. cancrivora* at 16S is *F. verruculosa*, which is restricted to the Lesser Sunda islands (green in Fig. 1b). Six frog legs from brand C were identified as *L. macrodon*, namely two haplotypes (SFL08–09) that belong to two distinct lineages bearing as much as 2.9% of 16S divergence. SFL08 was previously only retrieved in French and German supermarkets (Ohler and Nicolas 2017; Dittrich et al. 2017), and in the wild, it is closely related to populations from western Java (orange in Fig. 1c). SFL09 was previously found in populations from central Java (brown in Fig. 1c). The last frog leg from brand C corresponds to another *Limnonectes* species, namely *L. kadarsani* from the Lesser Sunda islands (dark green in Fig. 1c). The haplotype retrieved (SFL10) is new and closely matched that species, with substantial divergence from the closest relative *L. dammermani* distributed in the same region (light green in Fig. 1c).

**Fig. 2:**
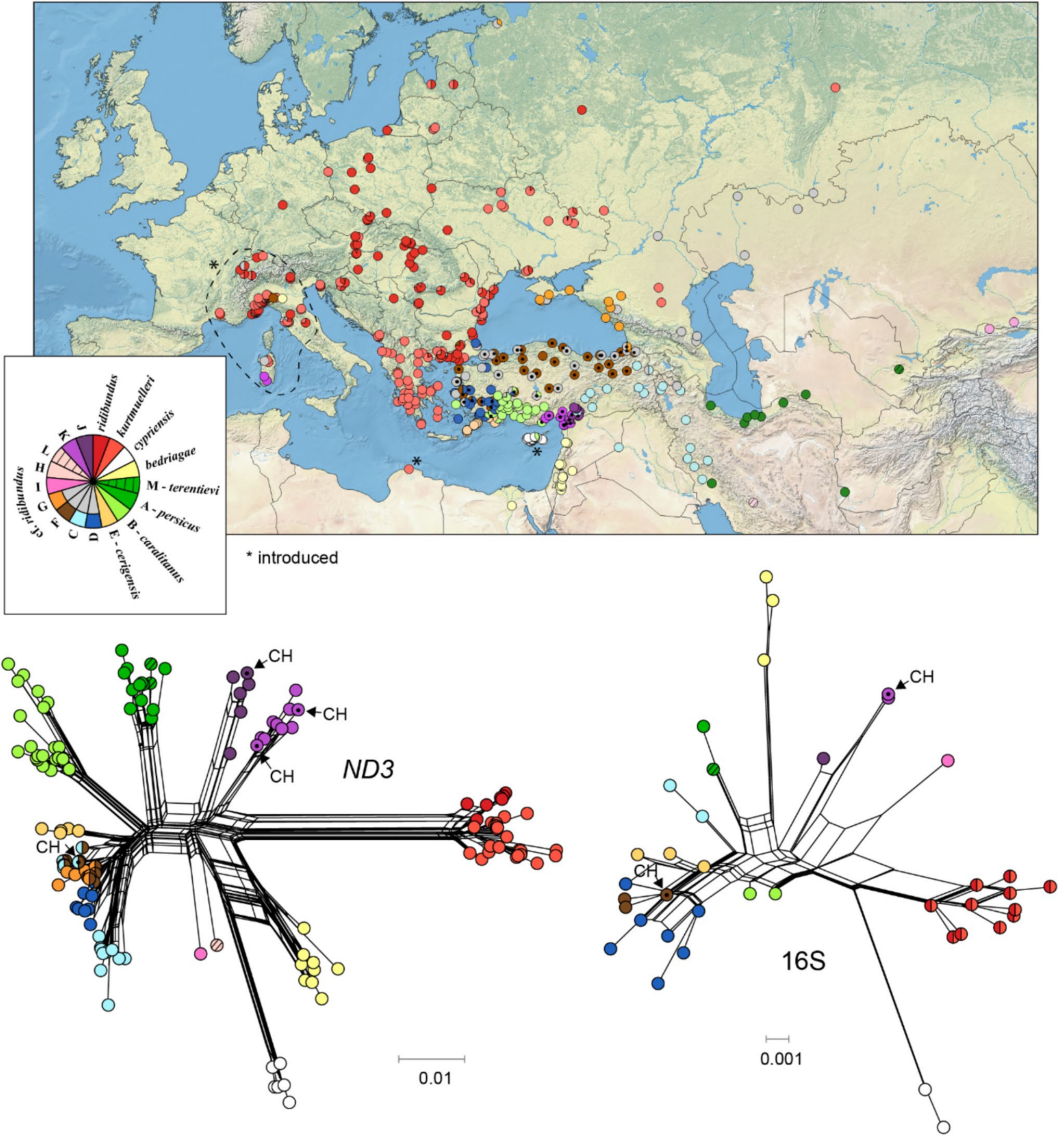
Geographic distribution (map) and relative divergence (networks) of the main mitochondrial lineages of *P. ridibundus* based 16S and *ND3*, according to Dufresnes et al. (2024). Dots emphasize the haplotypes retrieved in the Swiss frog legs and where these haplotypes segregate in the wild

The 16S sequences of brand E, which import live frogs transformed and sold as *Rana esculenta* [= *Pelophylax esculentus*], matched previously identified haplotypes attributed to *P. ridibundus*, and this was confirmed by additional sequencing of *ND3* (Table 1). This highly diverse species is composed of ~ 17 mitochondrial lineages of unsettled taxonomy distributed across Europe, the Near East and the Middle East (Fig. 2), as re-assessed by Dufresnes et al. (2024), whose haplotype and lineage labels are followed therein. Specifically, our sequences were identical or similar to haplotypes representative of three lineages given as *P. r.* cf. *ridibundus* J (a new haplotype ND3.197) and *P. r.* cf. *ridibundus* K (haplotypes 16S.036, ND3.127 and a new one ND3.196), which are naturally restricted to southern Turkey (light and dark purple in Fig. 2), as well as *P. r.* cf. *ridibundus* F (haplotypes 16S.026 and ND3.066; the latter being shared with the closely related lineage *P. r.* cf. *ridibundus* D), which is widely distributed across the northern half of Turkey (brown in Fig. 2). Identical haplotypes found in the wild are widespread across the ranges of the corresponding lineages (Fig. 2). Moreover, two of the three *P. ridibundus* lineages represented by the frog leg samples of brand E are known from introduced populations studied with 16S, *ND3*, and additional mitochondrial markers (Dufresnes et al. 2024 and references therein): lineage K in northern Italy, Sardinia, Switzerland and Ukraine; lineage F in northern Italy, Belgium, Luxemburg, and potentially Sardinia and Switzerland.

### Morphological analyses

**Fig. 3:**
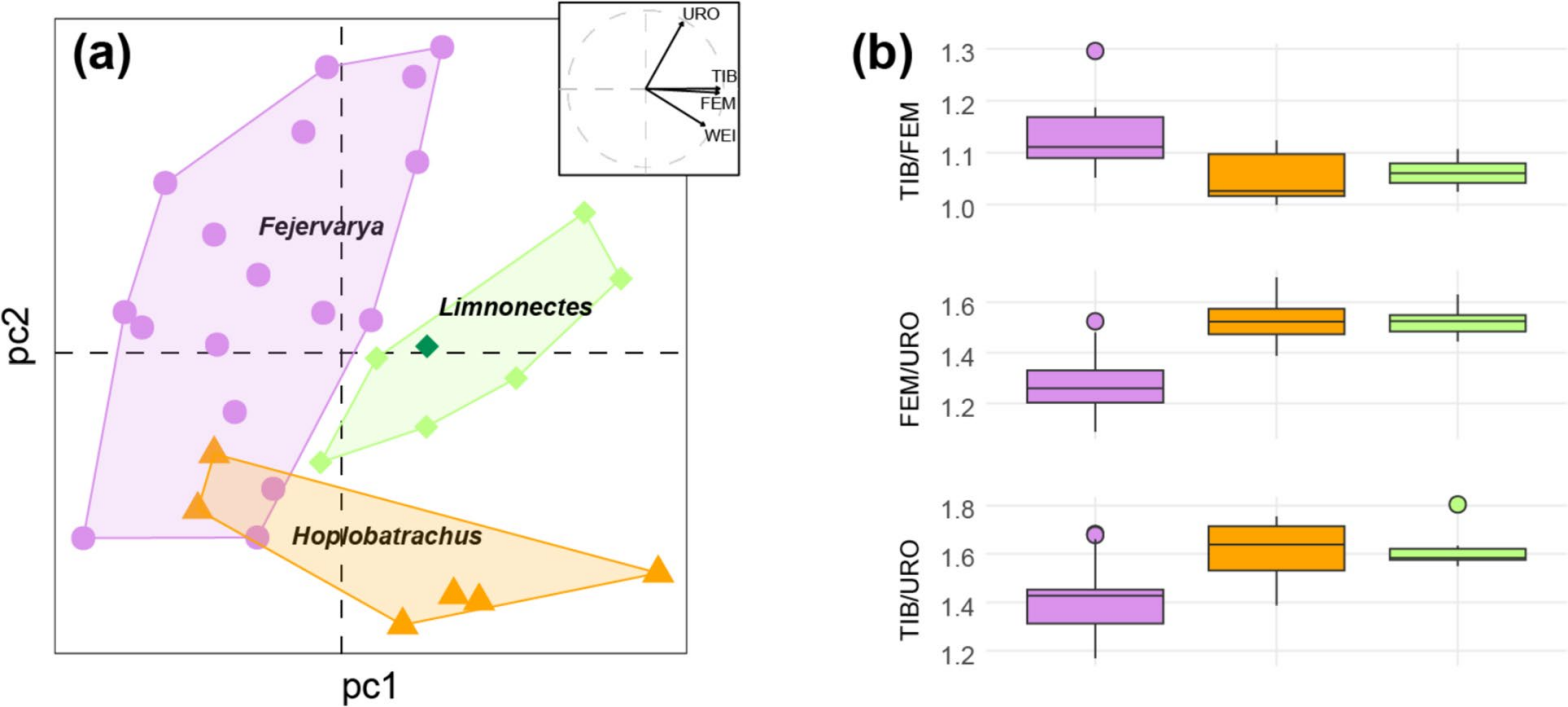
Comparisons of the morphology of frog legs between the three dicroglossid genera identified among the Swiss imports. **a** PCA based on four characters; convex hulls and symbols distinguish the three genera; for *Limonectes*, the *L. kardarsani* sample is shown in dark green. **b** Variation at the three measurement ratios considered

The frog legs attributed to the three dicroglossid genera (*Hoplobatrachus*, *n* = 6; *Fejervarya*, *n* = 17; *Limnonectes*, *n* = 7) barely overlapped in the morphospace (Fig. 3a). The single *L. kadarsani* specimen fell within the diversity of *L. macrodon* (Fig. 3a) and subsequent analyses were carried at the level of the genus. The three genera accordingly differ statistically in their general morphology (MANOVA; *F* = 15.6, *P* < 0.001), with significantly contributing variables being the femur length (FEM; *F* = 15.2, *P* < 0.001), the weight (WEI; *F* = 13.6, *P* < 0.001), the tibia length (TIB; *F* = 8.7, *P* = 0.001), but not the urostyle length (URO; *F* = 1.6, *P* = 0.22). All three measurement ratios analyzed were significantly different between genera, namely TIB/FEM (Kruskal–Wallis test, *χ*^*2*^ = 10.3, *P* = 0.006), TIB/URO (*χ*^*2*^ = 10.0, *P* = 0.01), and FEM/URO (*χ*^*2*^ = 17.3, *P* < 0.001). The ratios emphasize the longer tibia relative to the femur (high TIB/FEM), but the shorter tibia and femur relative to the urostyle (low TIB/URO and FEM/URO) of *Fejevarya* legs, as well as the shorter tibia relative to the femur (TIB/FEM) and the longer tibia relative to the urostyle (TIB/URO) of *Hoplobatrachus* legs (Fig. 3b). The LDA suggests that these frog legs may be reliably distinguished (entropy* R*^*2*^ = 0.94), with only one mismatch out of 30 samples: a *Fejervarya* misassigned to *Hoplobatrachus*.

## Discussion

Our study emphasizes the high diversity of species and phylogeographic lineages represented among the frog legs imported to Switzerland for human consumption, highlights shortcomings in package labelling, and demonstrates the possibility to distinguish the main Asian taxa involved without molecular analyses in future studies.

One of the most obvious although not unexpected finding is that only about half (55%) of the frog leg products we sampled were provided with the correct species information. Among them, the packages of *H. rugulosus* from Vietnam shared the 16S haplotype identified in the frog legs sold in Germany (Dittrich et al. 2017; Spielmann et al. 2018), thus potentially indicating the same trading network and geographic origin. Specifically, the *H. rugulosus* legs sold in Berlin supermarkets were given as “farmed” and were traced to the Mekong delta (Dittrich et al. 2017), which is home to intense aquaculture activities (Quoc 2012). The phylogeographic lineage this haplotype belongs to (“*H. rugulosus* south” in Fig. 1a) appears distributed in the southern part of the Indochinese Peninsula, but also in China, where its presence could be related to escapes from frog farms (Yu et al. 2015). The high mitochondrial diversity of *H. rugulosus* calls for taxonomic revisions and in depth phylogeographic analyses, which should also help assessing the impact of farming activities on the current diversity of populations.

While the Indonesian origin of the packages identified as “*F. cancrivora*” appears genuine, several of these frog legs belong to the distinct genus *Limnonectes*. *Fejervarya* and *Limnonectes* both include large size look-alike species that are collected indistinctively for the international trade in Indonesia (Altherr et al. 2011) and, therefore, are frequently retrieved among commercial imports to the EU (Dittrich et al. 2017; Ohler and Nicolas 2017). Incorrect species information may reflect misidentifications by the collectors and suppliers (Veith et al. 2000). Alternatively, *F. cancrivora* could be deliberately declared because it is a widespread and common species with lesser conservation concern than *L. macrodon* and *L. kadarsani*. The latter species have smaller ranges and face population declines potentially linked to over-harvesting (Ohler and Nicolas 2017). Anyhow, these composite packages reflect the extensive scale of the frog trade in Indonesia—*L. kadarsani* and *L. macrodon* inhabit different islands, and our *L. macrodon* samples correspond to two distinct phylogeographic lineages, thus indicating multiple origins. In fact, the 16S divergence (2.9%) of the *L. macrodon* lineages is higher than many valid amphibian species in Asia (Dufresnes and Litvinchuk 2022). This implies that the harvested populations of *L. macrodon* might represent several undocumented species with narrow geographic ranges, and which may thus face higher risks of extinction than presently assumed.

The unreliable traceability of frog legs imported from southeast Asia underscores the lack of regulation of the market and hinders the appreciation of its impact on natural populations by wildlife agencies. The available phylogeographic data suggests that the *Hoplobatrachus* and *Fejervarya* lineages harvested for the trade have been introduced outside their native range (Wostl et al. 2016; Yu et al. 2015) and could thus be involved in overlooked biological invasions. Proper information on the species exported from Indonesia is particularly important given that this country is the largest supplier of frog meat worldwide (3/4 of the EU imports; Altherr et al. 2022), but rather than being farmed (Kusrini and Alford 2006), animals are harvested directly and indiscriminately in the wild from multiple locations. Accordingly, many of the Indonesian frog legs sold in Germany and France are also mislabelled—but in a reverse fashion than what we report here for Switzerland, i.e., declared as *Limnonectes* but often consisting of *Fejervarya* (Dittrich et al. 2017; Ohler and Nicolas 2017). In the past, *L. macrodon* used to be a major source of frog legs but it now appears under-represented compared with other Indonesian species, suggesting a major decline (Ohler and Nicolas 2017). The enormous human pressure of its populations, which potentially threatens additional undescribed species, should hopefully motivate better regulations and monitoring to document the relative abundance of this frog among the harvested stocks.

A first step towards such regulations is to implement controls to determine the origin of imported stocks by forensic methods. Besides DNA barcoding, reliable identification may be achieved by analyzing isotope composition of the frog legs, which was shown to discriminate the three Asian genera frequently consumed and identified in the EU and Switzerland, namely *Hoplobatrachus*, *Fejervarya*, and *Limnonectes* (Dittrich et al. 2017). In parallel, we illustrate here that measuring a few simple anatomical characters may suffice for efficient discriminations among these genera, which requires only basic equipment (a digital calliper and a scale) and training. Extending sampling to additional brands and putative package sources should help build a reference database for future forensic applications.

Finally, brand E perpetuates the obsolete designation *Rana esculenta*, which was updated to *Pelophylax esculentus* nearly 20 years ago (Frost et al. 2006) and is used as such by taxonomic authorities dealing with amphibians (Frost 2025; Speybroeck et al. 2020), including wildlife authorities in Switzerland (Schmidt et al. 2023). One reason for this outdated name might be that the Swiss law for the protection of captive animals was enforced before these changes and accordingly lists water frogs as *Rana* sp. (Anonymous 2006). Anyhow, *Rana esculenta*/*Pelophylax esculentus* is inappropriate for the Turkish imports of brand E, because it refers to the hybrid between the pool frog *P. lessonae* and the marsh frog *P. ridibundus*, a form distributed across the temperate and continental regions of Europe but that is entirely absent from Turkey (Dufresnes and Mazepa 2020; Kurnaz 2020; Speybroeck et al. 2016). Accordingly, the DNA barcoding data identified the Turkish frog legs as *P. ridibundus*, the only *Pelophylax* species now recognized in Turkey. With no less than eight phylogeographic lineages in Anatolia alone (Akın et al. 2010; Dufresnes et al. 2024), *P. ridibundus* is a widespread and highly diverse species under which many taxa have recently been lumped (e.g., *P. r. kurtmuelleri*, *P. r. bedriagae*, *P. r. cypriensis*). We retrieved three different Anatolian lineages out of only four barcoded samples, which points to at least two regions of origins for the frog legs imported to Switzerland. One of them, the southern province of Adana, which is inhabited by the lineages labelled *P. r.* cf. *ridibundus* J and K (Fig. 2), is a hotspot of frog harvest for the international trade. A third of the annual exports of Turkey (which is itself the third most important supplier of the EU market; Altherr et al. 2022), corresponding to ~ 17 million individuals, originate from two river deltas of this area (Çiçek et al. 2021). The second source might be anywhere across the northern half of Turkey, where the haplotype we retrieved (from lineage *P. r.* cf. *ridibundus* F) has been widely reported (Fig. 2). Moreover, two of the lineages identified among the Turkish frog legs (*P. r.* cf. *ridibundus* F and K) were previously detected in the invasive populations of *P. ridibundus* in Western Europe (Bellati et al. 2023; Hollsbeck et al. 2008), including Switzerland, where some expanding clusters might have initiated from the vicinity of import companies (Dufresnes et al. 2018). Our results thus corroborate the partial responsibility of the frog leg industry in the ongoing invasions by this species, of which we are only now starting to gauge the extensive spatial extent (Dufresnes et al. 2024) and ecological impact (Denoël et al. 2022). This responsibility should be a prime argument to ban the export of live *Pelophylax* specimens in a strong and unified international response.

## Supplementary Information

Below is the link to the electronic supplementary material.

Supplementary file1 (XLSX 27 KB)

Supplementary file2 (XLSX 15 KB)

## Data Availability

All sequences used in this study (new and previously published) are publicly available on GenBank, with accession numbers provided in Table 1, in the Supporting Information (Appendix S1 and S2) and on Zenodo (10.5281/zenodo.10423702).
